# Immune Thrombocytopenia following COVID-19 Vaccine

**DOI:** 10.1155/2022/6013321

**Published:** 2022-06-25

**Authors:** Sonal Prasad, Roopam Jariwal, Moujidin Adebayo, Sara Jaka, Greti Petersen, Everardo Cobos

**Affiliations:** ^1^Ross University School of Medicine, Bridgetown, Barbados; ^2^Department of Internal Medicine, UCLA at Kern Medical Center, Bakersfield, CA 93306, USA

## Abstract

Several vaccines have been developed and are being administered against severe acute respiratory syndrome coronavirus 2 (SARS-CoV-2). Common side effects include fever, chills, headache, myalgia, and soreness at the injection site. However, some rare adverse effects have also been reported. We present a case of induced thrombocytopenia presenting with petechiae and mucosal bleeding which developed as an adverse response after first-dose administration of the Moderna COVID-19 vaccine.

## 1. Introduction

Immune thrombocytopenic purpura (ITP) is an acquired thrombocytopenia characterized by a platelet count less than 100 × 10^9^/L caused by autoantibodies against platelet antigens leading to increased platelet destruction and decreased platelet production [1]. Many vaccines such as influenza, varicella, measles, mumps, and rubella are known to induce ITP [2]. More recently, cases of thrombocytopenia following COVID-19 vaccination have been reported since the initiation of mass vaccinations. Two different types of immune thrombocytopenia are being reported which include vaccine-induced immune thrombocytopenia (VIT) and vaccine-induced immune thrombotic thrombocytopenia (VITT). The messenger RNA (mRNA) vaccines against SARS-CoV-2 are the first of their kind with different mechanisms of action. Understanding of the clinical manifestations in response to vaccination, their pathogenesis, and subsequent treatment of related thrombocytopenia is still evolving.

## 2. Case

**Figure 1:**
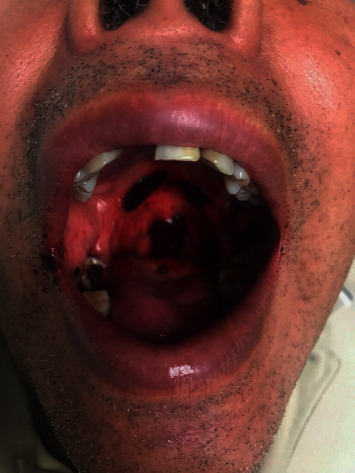
Significant gingival and oral mucosal bleeding.

A 58-year-old Hispanic male with hypertension and diabetes presented to our hospital for acute onset of mucosal bleeding, petechiae, and easy bruising. He denied any previous history of spontaneous bleeding or easy bruising. Physical examination was notable for diffuse petechiae along the arms, legs, and abdomen along with numerous oral lesions and gingival bleeding (Figure 1). He was noted to have a platelet count of 3 × 10^9^/L with all other cell lines within normal limits. In addition, the following labs were also within normal limits: prothrombin time, partial thromboplastin time, fibrinogen, d-dimer, bilirubin, lactate dehydrogenase, and haptoglobin. Coombs test to detect red blood cell antibodies was negative. Peripheral blood smear showed sparse platelets, no platelet clumping, and normal morphology of all other cells. Platelet antibodies were all negative. Viral studies for human immunodeficiency virus and hepatitis A, B, and C were all nonreactive. The antinuclear antibody test for autoimmune workup was negative. The platelet factor 4 antibody test was weakly positive, but the confirmatory serotonin-release assay was negative. Ultrasound of the abdomen showed hepatic steatosis and mild splenomegaly with the spleen measuring 12.6 cm in length. These tests were obtained to rule out disseminated intravascular coagulation, hemolytic uremic syndrome, thrombotic thrombocytopenic purpura, heparin-induced thrombocytopenia, and viral-induced thrombocytopenia. Further history taking elucidated that the patient had not had any recent upper respiratory infection or COVID-19 infection. COVID-19 test was negative. The patient had received his first dose of the Moderna COVID-19 vaccine on April 28, 2021; three weeks prior to the development of spontaneous bleeding. After a comprehensive and exhaustive workup of all well-known precipitants of ITP returned negative, a diagnosis of idiopathic thrombocytopenic purpura was made.

We treated the patient with high-dose dexamethasone 40 mg/day for four days and transfused five units of platelets, but his platelet count continued to be refractory with counts of 7 × 10^9^/L. He was then started on intravenous immunoglobulin (IVIG) 1 g/kg for two days. After two doses of IVIG, his platelet count improved to 39 × 10^9^/L, with the resolution of his mucosal bleeding, and he was discharged home. Five days later, the patient re-presented to our hospital with recurrent gingival bleeding, this time with a platelet count of 7 × 10^9^/L. He was transfused one unit of platelets, started on high-dose dexamethasone 40 mg/day for four days, and IVIG 1 g/kg for two days. Four days into admission, his platelet count improved to 41 × 10^9^/L, and he was again discharged home. Patient was not discharged on a tapering regimen of steroid medication. Five days after his second hospital discharge, he presented for a third time once more with gingival bleeding with a platelet count <3 × 10^9^/L. This time, we treated the patient with three platelet transfusions, two doses of IVIG 1 g/kg, as well as romiplostim—a thrombopoietin receptor agonist (TPO-RA)—starting at 1 mcg/kg and increasing by 1 mcg weekly, and fostamatinib—an inhibitor of spleen tyrosine kinase (SYK)—100 mg twice daily. The patient received a total of two doses of romiplostim with improvement in the platelet count to 18 × 10^9^/L and resolution of symptoms. He was then discharged home with fostamatinib 100 mg twice daily with a close follow-up in the hematology clinic. Ten days after hospital discharge, the patient's platelet count increased to 81 × 10^9^/L with no recurrence in bleeding. His most recent platelet count is 109 × 10^9^/L, and he is maintained on fostamatinib 100 mg twice daily. Patient refused to receive the second dose of the Moderna vaccine as well as any other vaccine type.

## 3. Discussion

With the widespread administration of COVID-19 vaccines to combat and curb the pandemic, adverse reactions have been reported including rare instances of thrombocytopenia and thrombosis. More specifically, there have been reported cases of both vaccine-induced immune thrombocytopenia (VIT) and vaccine-induced immune thrombotic thrombocytopenia (VITT). VITT presents within 4–30 days of vaccination with symptoms of thrombocytopenia and thrombosis including cerebral venous sinus thrombosis, splanchnic thrombosis [1], pulmonary embolism, and deep vein thrombosis. Significant laboratory findings in VITT include low fibrinogen, elevated d-dimer, and low platelets [3]. VIT has been noted to present with thrombocytopenia within 1–23 days with symptoms of bleeding, bruising, and petechiae [1]. Laboratory findings in VIT are only remarkable for severely low platelet count.

Clinical manifestations of VIT and VITT are similar to heparin-induced thrombocytopenia (HIT). In HIT, patients develop an antibody directed against endogenous platelet factor 4 (PF4) in complex with heparin, after exposure to heparin. This antibody activates platelets resulting in thrombosis. Recent reports show that patients with VIT and VITT secondary to Pfizer and Moderna COVID-19 vaccines have antibodies against PF4 on the enzyme-linked immunosorbent assay (ELISA) [4], a similar finding reported in HIT. Our patient presented with symptoms and laboratory findings concerning HIT, yet he had no prior exposure to heparin. As VIT rose amongst our differential diagnoses, we obtained an ELISA to see if there were antibodies against PF4. This test resulted as weakly positive. In HIT, a serotonin-release assay (SRA) can be used as a confirmatory test for antibodies against PF4—SRA performed on our patient's serum sample resulted negative. However, the confirmatory test does not seem to hold the same clinical significance in VIT and VITT as in HIT. Many cases report that only an ELISA test was performed, and no other confirmatory test was utilized to make the diagnosis of VIT/VITT. Although SRA is highly sensitive for HIT, one study showed evidence of patients with HIT who were SRA-negative [5]. Hence, SRA may not be needed to accurately make the diagnosis of VIT or VITT. However, as there are conflicting reports regarding the need for the confirmatory test, further investigation is required. In another article with positive cases of VITT, HIT, the ELISA was used to detect anti-PF4 antibodies and were positive; however, the rapid non-ELISA tests were negative. Hence, they recommend that it might be beneficial to repeat testing with a different ELISA method as they suggest that the antibodies present in these patients could have an epitope that is not recognized by the currently available assays [6].

There is confounding evidence whether VIT and VITT occur via the same mechanism or if they are the same reaction with a spectrum of manifestations. A review of the literature shows that VITT has been the predominantly reported thrombocytopenia in adenoviral vector-based vaccines such as AstraZeneca [1, 3, 4] and Johnson & Johnson [1], whereas VIT has mostly been reported in mRNA-based vaccines such as Pfizer and Moderna [4]. However, more research is needed to establish a correlation between the occurrence of VIT and VITT with the type of COVID-19 vaccine being administered.

Our patient received the Moderna vaccine and three weeks later presented with isolated thrombocytopenia which we ultimately diagnosed as VIT. We cannot be sure whether the patient had preexisting ITP but not by history, and there was no prior documentation. Of reported cases of VIT, most patients responded to treatment with corticosteroids and IVIG [7]. In one case report, a 22-year-old healthy male developed VIT after receiving the Pfizer vaccine and was treated with dexamethasone 40 mg/day for four days, a platelet transfusion, and IVIG 1 g/kg for two days which eventually led to resolution of the VIT [8]. Refractory cases of thrombocytopenia to both steroids and IVIG may benefit from a thrombopoietic agent. We treated our patient with multiple doses of steroids, IVIG, and platelet transfusions, yet his platelet count remained persistently low. The platelet count increased but declined soon afterwards. Due to his recurrent presentations and hospitalizations, we resorted to a combination of a TPO-RA—romiplostim—and a SYK inhibitor—fostamatinib. This drug combination was finally able to stabilize and increase our patient's platelet count. A case report published in the Journal of Blood Medicine utilized romiplostim alone as a second-line agent to success for VIT after steroids and IVIG were unsuccessful. With our review of the literature, the combination of TPO-RA and SYK inhibitors for treatment of refractory VIT has not been reported elsewhere. However, no specific algorithm has shown efficacy for treatment [6].

Our case is unique in that multiple treatment modalities were employed to increase platelet count with multiple rounds of steroids, platelet transfusions, and IVIG, despite which, our patient's platelet count remained low. Finally, treatment with TPO-RA and SYK inhibitors was able to stabilize his platelet count.

## 4. Conclusion

COVID-19 vaccine-induced ITP is a rare adverse effect that needs continued surveillance. Since the mechanism of COVID-19 VIT is unclear, it is worthwhile to further investigate the use of TPO-RA and SYK inhibitors as treatment options. Vaccination against SARS-CoV-2 is imperative to control the global pandemic, and clinicians need to be aware of and have a low index of suspicion on making the diagnosis of VIT or VITT. It is important to acknowledge that some patients may not respond to the standard accepted treatment with steroids and IVIG. Cases similar to ours may benefit from therapy with TPO-RA and the SYK inhibitor when first-line therapy with steroids and IVIG are unsuccessful.
